# Modeling RET-Rearranged Non-Small Cell Lung Cancer (NSCLC): Generation of Lung Progenitor Cells (LPCs) from Patient-Derived Induced Pluripotent Stem Cells (iPSCs)

**DOI:** 10.3390/cells12242847

**Published:** 2023-12-15

**Authors:** Paul Marcoux, Jin Wook Hwang, Christophe Desterke, Jusuf Imeri, Annelise Bennaceur-Griscelli, Ali G. Turhan

**Affiliations:** 1 INSERM UMR-S-1310, Université Paris Saclay, 94800 Villejuif, France; paul.marcoux@inserm.fr (P.M.); jinwook.hwang@inserm.fr (J.W.H.); christophe.desterke@gmail.com (C.D.); jusuf.imeri@inserm.fr (J.I.); abenna@hotmail.fr (A.B.-G.); 2 Faculty of Medicine, Paris-Saclay University, 94270 Le Kremlin Bicetre, France; 3 APHP Paris Saclay, Department of Hematology, Hôpital Bicêtre, 94270 Le Kremlin Bicetre, France; 4 Center for IPSC Therapies, CITHERA, INSERM UMS-45, Genopole Campus, 91100 Evry, France; 5 APHP Paris Saclay, Department of Hematology, Hôpital Paul Brousse, 94800 Villejuif, France

**Keywords:** NSCLC, patient derived, iPSCs, RET, LPC differentiation, cancer, model, pralsetinib

## Abstract

REarranged during Transfection (RET) oncogenic rearrangements can occur in 1–2% of lung adenocarcinomas. While RET-driven NSCLC models have been developed using various approaches, no model based on patient-derived induced pluripotent stem cells (iPSCs) has yet been described. Patient-derived iPSCs hold great promise for disease modeling and drug screening. However, generating iPSCs with specific oncogenic drivers, like *RET* rearrangements, presents challenges due to reprogramming efficiency and genotypic variability within tumors. To address this issue, we aimed to generate lung progenitor cells (LPCs) from patient-derived iPSCs carrying the mutation *RET^C634Y^*, commonly associated with medullary thyroid carcinoma. Additionally, we established a *RET^C634Y^* knock-in iPSC model to validate the effect of this oncogenic mutation during LPC differentiation. We successfully generated LPCs from *RET^C634Y^* iPSCs using a 16-day protocol and detected an overexpression of cancer-associated markers as compared to control iPSCs. Transcriptomic analysis revealed a distinct signature of NSCLC tumor repression, suggesting a lung multilineage lung dedifferentiation, along with an upregulated signature associated with *RET^C634Y^* mutation, potentially linked to poor NSCLC prognosis. These findings were validated using the *RET^C634Y^* knock-in iPSC model, highlighting key cancerous targets such as *PROM2* and *C1QTNF6*, known to be associated with poor prognostic outcomes. Furthermore, the LPCs derived from *RET^C634Y^* iPSCs exhibited a positive response to the RET inhibitor pralsetinib, evidenced by the downregulation of the cancer markers. This study provides a novel patient-derived off-the-shelf iPSC model of RET-driven NSCLC, paving the way for exploring the molecular mechanisms involved in RET-driven NSCLC to study disease progression and to uncover potential therapeutic targets.

## 1. Introduction

Lung cancer is the second most prevalent cancer worldwide with over 2.2 million new cases reported in 2020 [1]. Non-small cell lung cancer (NSCLC) accounts for approximately 85% of these cases, with adenocarcinoma being the most common subtype among all lung cancers, comprising 40% of cases [2,3]. The classification of lung adenocarcinomas into molecular subtypes is determined by specific molecular alterations that contribute to cancer initiation and progression [4]. Many of these oncogenic drivers are receptor tyrosine kinases (RTKs) that regulate intracellular signaling pathways [5].

One of these RTK, REarranged during Transfection (RET), has been extensively studied in NSCLC. RET transmits a proliferative signal in the presence of its co-receptor GDNF (glial cell line derived neurotrophic factor) family receptor alpha-1 (GFRα1) and in response to GDNF-ligands families (GLF). Recent studies have revealed the presence of *RET* rearrangements in 1–2% of cases of lung adenocarcinoma [6]. Patients with RET-fusion positive NSCLC are mostly young-never smokers [7]. RET fusions lead to the activation of downstream signaling pathways such as STAT3 and RAS-MAPK involved in cell proliferation and survival, thus promoting tumor growth [8,9,10]. RET fusion-positive lung carcinomas exhibit poorer differentiated tumors compared to those with ALK or EGFR alterations [11]. Moreover, previous evidence has indicated that RET signaling plays a significant role in drug resistance, including resistance to EGFR TKIs and emerging KRAS^G12C^ inhibitors in NSCLC [12,13]. Finally, *RET*-rearranged patients typically exhibit low levels of PD-L1 expression and a low tumor mutational burden, and they tend to have unfavorable outcomes when treated with immunotherapies [14]. These data show that *RET* rearrangements define a distinct molecular and clinicopathological subtype of NSCLC. Therefore, the development of a *RET*-rearranged lung cancer model would be highly valuable to investigate the unique characteristics of this disease and identify novel therapeutic targets.

Several models of *RET*-rearranged lung cancer have been developed during previous years. These models are based on cancer cell lines [15], genetically engineered mouse models expressing KIF5B-RET fusion protein [16], or PDX-derived lung adenocarcinoma cells harboring KIF5B-RET fusion [17].

iPSCs have been used previously to model several types of malignancies including leukemia [18,19,20], hereditary cancers such as Li-Fraumeni syndrome [21] kidney cancer [22], and hereditary retinoblastoma [23]. The role of oncogenic KRAS has been studied in alveolar cells derived from human iPSCs expressing Dox-inducible KRAS^G12D^, revealing a down-regulation of maturation markers in alveolar cells expressing KRAS^G12D^ with upregulation of progenitor and developmental markers [24]. However, this highly interesting model used normal donor-derived iPSCs to study the effect of the expression of oncogenic KRAS^G12D^ in alveolar epithelial cells. Currently, no lung cancer model based on patient-derived induced pluripotent stem cells (iPSCs) has yet been developed. Such a model could constitute a valuable asset to study RET-driven NSCLC. Indeed, patient-derived iPSC models capture the unique genetic characteristics of individual patients and provide a more accurate representation of the disease biology compared to traditional cell lines [25]. By facilitating disease modeling and the creation of patient-specific organoids, patient-derived iPSCs enable the investigation of cancer development, high-throughput drug screening, and target discovery, paving the way for remarkable progress in these critical domains [22].

However, generation of patient-derived iPSCs from NSCLC patients carrying specific oncogenic drivers, such as *RET* rearrangements, presents significant challenges. Indeed, most reprogramming protocols are optimized for cells that are easily available and more efficient to reprogram, with high proliferation rate and chromatin accessibility, such as peripheral blood mononuclear cells (PBMC), mesenchymal stem cells, fibroblast, etc. [26]. Additionally, the low efficiency of reprogramming, coupled with the high genotypic variability within tumors, further complicates the generation of iPSCs with specific mutations of interest [27]. Finally, like other cancer cells, the reprogramming of NSCLC cells into iPSCs is impeded by various barriers, including genetic alterations and epigenetic memory [18]. Although, the generation of iPSCs carrying hereditary mutations is a more attainable objective due to their presence in all cells of an individual, it enables the utilization of existing reprogramming protocols [21]. Several studies have already used patient-derived iPSCs carrying mutations, such as *p53* or *RB1* mutations, to elucidate mechanisms related to cancer [23,28]. However, to the best of our knowledge, there are presently no NSCLC patient-derived iPS cell lines and therefore no model of a patient-derived-iPSC lung cancer model.

To tackle this challenge, we tested whether we could generate lung progenitor cells (LPCs) accurately recapitulating the characteristics of *RET*-rearranged NSCLC from a patient-derived iPSC carrying the *RET^C634Y^* point mutation. *RET^C634Y^* mutation is commonly associated with medullary thyroid carcinoma (MTC) and results in RET dimerization in the absence of its ligands, leading to the autophosphorylation of its tyrosine kinase domains. The constitutive activation of the RET pathway is equivalent to the consequences of *RET* rearrangements observed in NSCLC [29,30,31]. We also generated a *RET^C634Y^* knock-in iPSC to validate the effect of the mutation on iPSC-derived lung progenitors. Therefore, this work aimed to establish the suitability of iPSCs carrying RET point mutations as the first model of patient-derived iPSCs RET-driven NSCLC.

Using a 16-day protocol [32], we successfully generated lung progenitors from patient-derived iPSCs harboring the *RET^C634Y^* mutation (iRET^C634Y^) and its CRISPR-corrected isogenic control iPSC (iRET^CTRL^). Notably, progenitors derived from iRET^C634Y^ exhibited an overexpression of cancer-associated markers as compared to WT progenitor derived from iRET^CTRL^. Transcriptomic analysis uncovered a distinctive repressed signature of NSCLC that was dependent on the *RET^C634Y^* mutation, indicating lung multilineage dedifferentiation. Additionally, the upregulated signature associated with *RET^C634Y^* mutation could potentially be linked to poor prognosis for NSCLC. These findings were further validated by employing a knock-in of the *RET^C634Y^* mutation in WT iPSCs (PB68-RET^C634Y^ and PB68-WT). In both approaches, key targets associated with poor prognostic outcomes, namely *PROM2* and *C1QTNF6*, were found to be upregulated by the *RET^C634Y^* mutation. Finally, LPCs derived from iPSCs carrying the *RET^C634Y^* mutation demonstrated a positive response to the RET inhibitor pralsetinib, as evidenced by the downregulation of these cancer markers.

## 2. Materials and Methods

### 2.1. Generation of iPSCs

The iPSC line PB68-WT was generated from peripheral blood mononuclear cells (PBMCs) obtained from the cord blood of healthy donors according to the Declaration of Helsinki. Cells were reprogrammed by non-integrative Sendai viral transduction. Pluripotency was characterized by FACS and teratoma assays. PB68-RET^C634Y^ was generated from the iPSC PB68-WT using lentiviral transduction described in a previous study [33]. Briefly, we used Lenti-X 293T as a packaging cell line and psPAX2.2, and pMD2.G as packaging vector and envelope vector, respectively. The plasmid RET^C634Y^ was purchased from VectorBuilder (Guangzhou, China). Generation of RET mutated iPSC iRET^C634Y^ and its isogenic CRIPSR corrected control iRET^CTRL^ were previously described [34,35].

iPSCs were cultured in feeder-free condition in Geltrex coated dishes (A1413201; ThermoFisher Scientific, Illkirch, France) and fed daily with Essential 8 flex Medium (A2858501; ThermoFisher Scientific, Illkirch, France). iPSCs were passaged twice a week with EDTA dissociation (0.5 mM).

### 2.2. Generation of Lung Progenitor Cells

This procedure was adapted from the work of Leibel and colleagues [32]. The protocol involves the stepwise differentiation of iPSCs to lung progenitor cells (LPCs). iPSCs were seeded at 55–70% confluency in 6-well plates coated with Geltrex the day before definitive endoderm induction (DE). DE induction medium is composed of RPMI1640 (11875093; Gibco, Illkirch, France) supplemented with Glutamax (35050061; Gibco, Illkirch, France), B27 (12587010; ThermoFisher, Illkirch, France), Pen/Strep (15140-122; Gibco, France), HEPES 1% (15630-080; Gibco, Illkirch, France), 100 ng/mL Human activin A (338-AC; R&D Systems, Lille, France) and 5 µM CHIR99021 (72054; Stemcell Technology, Grenoble, France). DE induction medium was replaced daily for 3 days. On day +4, the medium was changed and replaced daily until day +6 with anterior foregut endoderm (AFE) induction medium which is serum free basal media supplemented with 10 μM SB431542 (1614; Tocris Bioscience, Bristol, UK) and 2 μM dorsomorphin (72102; Stemcell, Grenoble, France). Serum free basal medium is composed of 75% IMDM+Glutamax (31980030; Gibco, Illkirch, France) and 25% Ham’s F12 (11765054; Gibco, France) complemented with B27, N2 (17502048; ThermoFisher, Illkirch, France), Pen/Strep, 50 mg/mL L-Ascorbic acid 2-phosphate (A8960; Sigma-Aldrich, Saint-Quentin-Fallavier, France), 500 μg/mL monothioglycerol (M6145; Sigma-Aldrich, Saint-Quentin-Fallavier, France), 7,5% BSA (15260-037; Gibco, Illkirch, France). On day +7, the AFE medium was aspirated, and replaced by LPC induction medium and changed every two days. LPC induction medium is composed of serum free basal medium complemented with 10 ng/mL human BMP4 (78211; Stemcell, Grenoble, France), 0.1 μM all-trans retinoic acid (72262; Stemcell, Grenoble, France), and 3 μM CHIR99021.

### 2.3. RNA Extraction, Reverse Transcription, and qRT-PCR

Total intracellular RNA was extracted using RNeasy Mini Kit (74104; Qiagen, Hilden, Germany) and 1 µg was reverse transcribed using a reverse transcription (RT)-PCR kit (Superscript III 18080-44; ThermoFisher Scientific, Illkirch, France). An aliquot of cDNA was used as a template for qRT-PCR analysis using a fluorescence thermocycler (ThermoFisher Scientific QuantStudio 3TM) with FastStart Universal SYBR Green (04913914001, Roche, Vilnius, Lithuania) DNA dye. The primer sequences used for qRT-PCR are listed in the Supplementary Table S1. Relative expression was normalized to the geometric mean of housekeeping gene expression and was calculated using the 2^−ΔΔCt^ method.

### 2.4. Immunofluorescence Staining

LPCs were washed with phosphate-buffered saline (PBS) fixed with 4% formaldehyde in PBS for 60 min, permeabilized with 0.2% Triton X-100 (Sigma-Aldrich, Saint-Quentin-Fallavier, France) in PBS and blocked with 10% serum. Primary antibodies were diluted in PBS 10% serum at the following concentrations: TP63 (1:100, ab124762; Abcam, Cambridge, UK) and Phospho-RET (Tyr1096) (1:100, PA5-105796; Thermo Fisher Scientific, Illkirch, France). Samples were incubated with secondary antibodies in antibody dilution buffer, then washed in PBS. Nuclei were labeled with DAPI (D9542; Sigma-Aldrich, Saint-Quentin-Fallavier, France) mounting medium. Visualization and capture were performed with a Leica confocal microscope and LAS AF software (v3.2). 

### 2.5. RNA-Sequencing Experiments

iPSCs and iPSC-derived LPC samples were processed for transcriptome triplicate experiments. Before the preparation of the sequencing library, the quality of the RNAs was checked with bioanalyzer with an average RIN per sample of 9.6. Starting from 10 to 100 ng of total RNA, molecular library of sequencing (Illumina) preparation was conducted for paired end sequencing focused on 3′ coding ends of transcripts. A minimum of ten million of reads were sequenced by sample on GENOMIC platform from Cochin Institute (Paris, France).

### 2.6. RNA-Sequencing Analyses

Paired-end FASTQ files were aligned on human genome version Ensembl release 101, Homo sapiens GRCh38 with STAR algorithm version (v2.7.6a) in two pass mode [36]. Transcript count was counted with RSEM algorithm version (v1.3.1) [37]. Transcript normalization and differential expressed gene analysis was performed with DeSeq2 R package version 1.34.0 in R environment version 4.1.3 [38].

### 2.7. Transcriptome Datasets

Transcriptome data of NSCLC samples and normal lung adjacent tissue from frozen sampling of Gene Expression Omnibus (GEO) dataset GSE44077 [39] were collected at this address https://www.ncbi.nlm.nih.gov/geo/query/acc.cgi?acc=GSE44077 (accessed on 12 May 2022). This transcriptomic analysis was performed with Affymetrix Human Gene 1.0 ST Array technology and annotated with the corresponding platform GPL6244 https://www.ncbi.nlm.nih.gov/geo/query/acc.cgi?acc=GPL6244 (accessed on 12 May 2022).

### 2.8. TCGA RNA-Sequencing of Lung Adenocarcinoma Tumors

Lung adenocarcinoma tumor transcriptome dataset from The Cancer Genome Atlas (TCGA) consortium [40] was accessed through CBioPortal web tool [41]. This cohort of transcriptome is composed of 510 lung tumors from patients with a median age of 66 years old (range from 33 to 88 years).

### 2.9. Bioinformatics Analysis

Bioinformatics analyses were performed with R version 4.1.3. Unsupervised principal component analysis (PCA) was carried out with prcomp R base function and drawn with autoplot function from ggforitfy R-package version 0.4.14. Microarray transcriptome differentially expressed gene analysis was conducted with limma R-package version 3.50.3. Expression heatmaps were drawn with pheatmap R-package version 1.0.12 and clustering was conducted with the parameters clustering distance = “euclidean” and clustering method = “complete”. Functional enrichment was performed by over representative analysis through two distinct webtool applications: Enrichr [42] and Toppgene suite [43]. These functional enrichment analyses were carried out with distinct databases: Gene Ontology [44], DisGeNET [45] and Co-expression Lung Atlas through GeneSigDB [46] and MsigDb [47]. Functional enrichment networks were built with Cytoscape standalone application version 3.6.0 [48]. Barplots were drawn with ggplot2 R-package 3.3.6 [49]. Iterative loop of univariate survival Cox model on expression of selected markers was performed with loopcolcox_1.0.0 R-package https://github.com/cdesterke/loopcolcox (accessed on 8 February 2023). Log rank survival analysis at univariate and multivariate levels was performed with survival R-package version 3.5-0. The expression risk score was computed with the sum of the mathematical products between the Cox beta coefficient and the expression of the selected molecular markers. The threshold on risk score and Kaplan–Meier graph were performed with survminer R-package version 0.4.9. Calibration of the Cox multivariable model was performed by bootstrapping with rms R-package version 6.4-1 and survival nomogram was drawn with regplot R-package version 1.1.

## 3. Results

### 3.1. iRET^CTRL^ and iRET^C634Y^ iPSCs Can Be Successfully Differentiated into Lung Progenitor Cells

To assess the potential of patient-derived iPSCs harboring inherited mutations as an accurate model of RET-driven NSCLC, we employed an iPS cell line derived from a patient carrying *RET^C634Y^* mutation (iRET^C634Y^) who developed medullary thyroid carcinoma (MTC) [34] Additionally, we included an isogenic CRISPR/Cas9-corrected iPSC line (iRET^CTRL^) as a control [35]. This model has already proven to be valuable in investigating the RET-activation related mechanisms [33].

**Figure 1:**
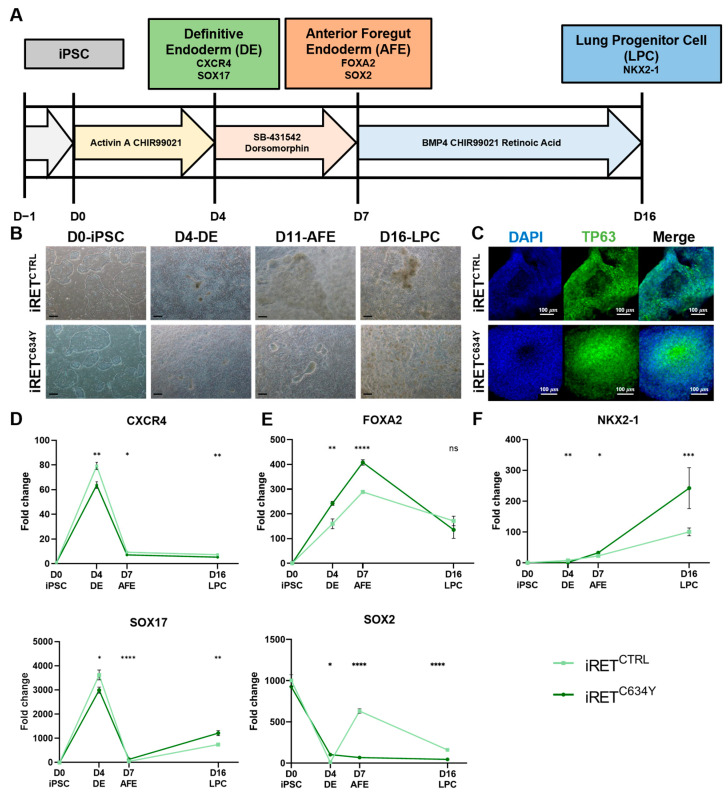
Generation of lung progenitor cells (LPCs) from iRET^C634Y^ is associated with the overexpression of FOXA2 and NKX2-1. (**A**) Schematic representation of the differentiation protocol from iPSC to NKX2-1^+^ lung progenitor cells (LPCs). (**B**) Morphology of *RET^C634Y^* mutated iPSC (iRET^C634Y^) and its isogenic CRISPR control (iRET^CTRL^) during LPC differentiation at definitive endoderm (DE), anterior foregut endoderm (AFE), and LPC stages. Magnification 10×; scale bar 100 μm. (**C**) Immunostaining of LPCs derived from iRET^C634Y^ and iRET^CTRL^ iPSCs showing the expression of TP63 (green), DAPI (blue) or merged. (**D**–**F**) Expression of the differentiation markers specific to each stage; (**D**) DE, (**E**) AFE, and (**F**) LPC; quantified by qRT-PCR. Fold change (2^−ΔΔCt^) was normalized to iPSC stage. Differentiation experiments were performed three times for each condition. *p*-values were calculated at each stage using a two-tailed Student’s *t*-test. ns, not significant; * *p* < 0.05; ** *p* < 0.01; *** *p* < 0.001; **** *p* < 0.0001.

These two iPS cell lines were differentiated into NKX2-1^+^ lung progenitor cells (LPCs) with a 16-day protocol [32]. The process involves enzymatic dissociation of iPSCs and their differentiation into LPC following sequential steps (Figure 1A). The first step is the induction of the definitive endoderm (DE) expressing *CXCR4* and *SOX17* [50], and then the generation of anterior foregut endoderm (AFE), characterized with the expression of *FOXA2* and *SOX2* [51]. Finally, the cells are specified into LPCs (Figure 1A) [52].

Phase-contrast imaging during the differentiation of both iRET^CTRL^ and iRET^C634Y^ iPSC differentiation revealed expected morphology at each stage for respective cell types as compared to previously published data, thus showing a typical morphology consistent with an ongoing differentiation (Figure 1B) [32]. To further confirm the phenotype of the cells obtained at day +16, an immunostaining for TP63 was performed. Both NKX2-1 and TP63 were shown to be expressed upon differentiation of pluripotent stem cells towards LPCs [53]. TP63 is a marker for basal cells in the human airway epithelium, which are multipotent stem cells involved in epithelial repair and regeneration [54]. Immunofluorescence staining demonstrated that cells derived from both iPSCs expressed TP63, indicating a successful differentiation into LPCs (Figure 1C).

### 3.2. Generation of LPCs from iRET^C634Y^ Is Associated with the Overexpression of Cancer-Related Markers and a Delay of Differentiation

To gain deeper insights into the processes occurring during LPC differentiation, qRT-PCR analyses were performed to assess the expression levels of stage-specific markers at various time points. Specifically, the characteristic markers for each stage, namely *CXCR4* and *SOX17* for DE, *FOXA2* and *SOX2* for AFE, and *NKX2-1* for LPC, were examined at day +4 (DE), day +7 (AFE), and day +16 (LPC). (Figure 1D). During the differentiation process of both iRET^CTRL^ and iRET^C634Y^ cell lines, expression levels of *CXCR4* and *SOX17* peaked at day +4, with a fold change of 60 and 3000, respectively, were compared to the expression levels in iPSCs (Figure 1D). Subsequently, their expression declined during the AFE stage and remained consistently low until the completion of differentiation. Interestingly, on day +4, the upregulation of *CXCR4* and *SOX17* was significantly higher in iRET^CTRL^ as compared to iRET^C634Y^.

The expression of *FOXA2* was increased during the first day of the differentiation and reached its maximum level at the AFE stage before decreasing at the LPC stage (Figure 1E). At the DE and AFE stages, *FOXA2* was significantly overexpressed in iRET^C634Y^ as compared to iRET^CTRL^. Interestingly, FOXA2 transcription factor is known to be upregulated in KIF5B-RET fusion adenocarcinomas through RET downstream signaling pathways such as ERK and AKT [55]. Hence, it is plausible that *RET^C634Y^* could upregulate *FOXA2* expression similarly during the differentiation of iPSC-derived LPCs.

*SOX2* is a pluripotency marker and is highly expressed in undifferentiated iPSCs (D0) (Figure 1E). As the iPSCs undergo differentiation, the expression of *SOX2* diminished in the initial days. Intriguingly, during the AFE stage, *SOX2* is re-expressed solely in iRET^CTRL^ cells and not in iRET^C634Y^ cells, before declining once more during the LPC stage. During the AFE stage, *SOX2* was shown to regulate the emergence of lung basal cells [56], consequently, its absence could potentially result in a differentiation defect associated with the *RET^C634Y^* mutation.

The expression of *NKX2-1* exhibited a consistent increase throughout the entire differentiation process, reaching its peak at the LPC stage (Figure 1F). Interestingly, LPCs derived from iRET^C634Y^ demonstrated a three-fold higher expression of *NKX2-1* compared to iRET^CTRL^-derived LPCs. NKX2-1 serves as a marker for LPC differentiation; however, it is also associated with cancer [57], particularly in lung adenocarcinoma where it is highly expressed [58,59]. Therefore, the overexpression of NKX2-1 in iRET^C634Y^-derived LPCs could potentially be linked to the formation of cancerous tissues.

Hence, the *RET^C634Y^* mutation appears to be linked to the upregulation of cancer-related markers and may be associated with a delay in the differentiation process, which is a characteristic feature of RET-driven NSCLC [11].

### 3.3. RET^C634Y^-Dependent Gene Signature during iPSC-Derived LPC Differentiation Predicts a Major Transcriptional Repression in NSCLC

**Figure 2:**
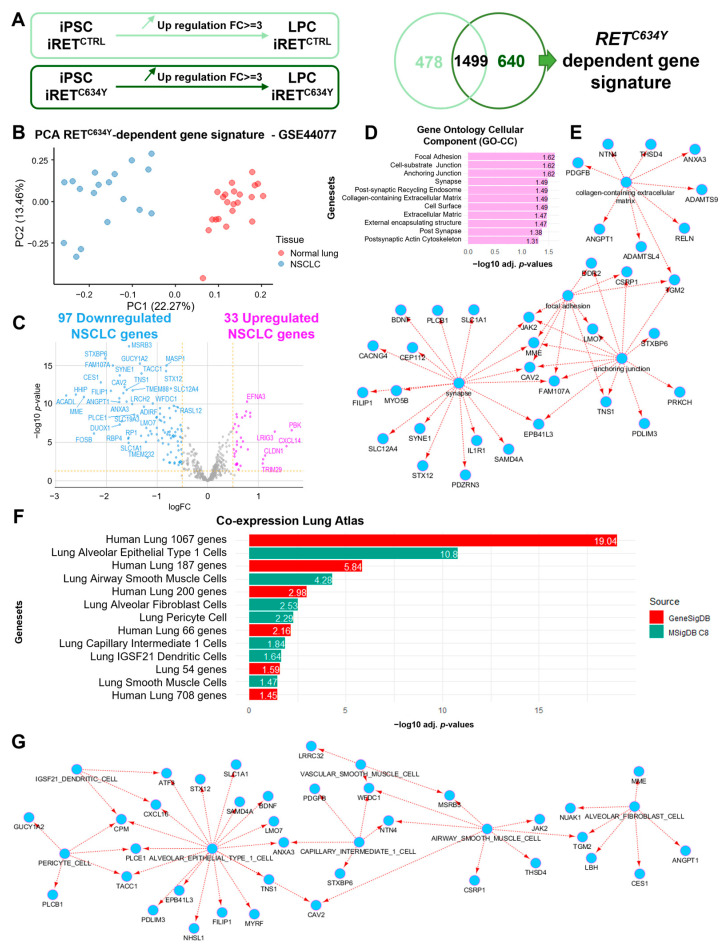
*RET^C634Y^*-dependent gene signature during iPSC-derived LPC differentiation predicts a major transcriptional repression in NSCLC associated with a lung multilineage dedifferentiation. (**A**) Method for analyzing the *RET^C634Y^*-dependent signature during iPSC-derived LPC differentiation. (**B**) Unsupervised principal component analysis based on *RET^C634Y^*-dependent gene signature can stratify tumoral and normal lung adjacent samples from GSE44077 transcriptome dataset. (**C**) Volcano plot of differential expressed gene analysis between tumor and lung adjacent tissue of GSE44077 restricted to *RET^C634Y^*-dependent gene signature (filter fixed over 0.5 log2 of fold change). (**D**) Barplot of functional enrichment performed with *RET^C634Y^*-dependent repressed signature on Gene Ontology Cellular Component (GO-CC) database. (**E**) Functional enrichment network highlighting the implication of connected components like focal adhesion, anchoring junction, and synapse in *RET^C634Y^*-dependent repressed signature in NSCLC tumors. (**F**) Barplot of functional enrichment performed on Co-expression Lung Atlas database with *RET^C634Y^*-dependent repressed NSCLC signature. (**G**) Functional enrichment network identifying a lung multilineage implication of *RET^C634Y^*-dependent repressed signature in NSCLC.

To evaluate the effect of *RET^C634Y^* mutation on transcriptional regulation during iPSC-derived LPC differentiation, whole transcriptome sequencing was performed in triplicate for iRET^C634Y^ and iRET^CTRL^ at both iPSC and LPC stages. During LPC differentiation, a total of 1977 and 2139 genes were found to be overexpressed in iRET^CTRL^ and iRET^C634Y^, respectively. The comparison between these two gene lists was performed and only 640 genes specifically overexpressed during iRET^C634Y^ LPC differentiation were retained constituting a specific *RET^C634Y^*-dependent gene signature (Figure 2A). To estimate the validity of this patient-derived iPSC NSCLC model and the influence of the *RET^C634Y^* mutation, *RET^C634Y^*-dependent gene signature was used to perform unsupervised analysis of NSCLC transcriptome data as compared to adjacent normal lung tissues. Principal component analysis performed (PCA) on GSE44077 transcriptome dataset revealed a good stratification of NSCLC tumor samples as compared to normal lung sample on the first principal axis based on *RET^C634Y^*-dependent gene signature (Figure 2B). Among the 640 *RET^C634Y^*-dependent genes, 97 genes were found to be significantly suppressed in tumors (Supplementary Table S2), while 33 genes were significantly upregulated (Supplementary Table S3). Supervised gene expression analysis restricted to *RET^C634Y^*-dependent gene signature highlighted a major differentiation inhibitory signature in NSCLC tumor samples as compared to normal lung tissue samples (Figure 2C). These results suggest that *RET^C634Y^*-dependent gene signature can predict a set of repressed genes in NSCLC.

### 3.4. RET^C634Y^-Dependent Inhibitory Signature in NSCLC Identifies a Lung Multilineage Dedifferentiation

*RET^C634Y^*-dependent repressed gene signature in NSCLC (Supplementary Table S2) was validated to effectively stratify NSCLC tumor samples from normal lung tissues through unsupervised clustering (Supplementary Figure S1A) as well as unsupervised PCA (Supplementary Figure S1B). Functional enrichment of these repressed genes, performed on the Gene Ontology Cellular Component (GO-CC) database, revealed their implication mainly in focal adhesion, anchoring junction, and synapse (Figure 2D,E). These results suggest that RET related transcriptional repression occurring in NSCLC could disrupt epithelial cell fate and matrix adhesion.

Functional enrichment performed on Co-expression Lung Atlas through GeneSigDB database confirmed that *RET^C634Y^*-dependent repressed gene signature in NSCLC samples was found to be affecting other human lung bulk signatures (red bars, Figure 2F). A comprehensive single-cell atlas of the normal human lung was generated, revealing a distinct gene signature for each lung cell subpopulation [60]. Enrichment based on this atlas reveals that RET-dependent repressed signature in NSCLC may affect several normal lung cell subpopulations such as type I alveolar epithelial cells (AT1s), airway smooth muscle cells, alveolar fibroblasts, pericytes, IGSF21 positive dendritic cells, and vascular smooth muscle cells (green bars, Figure 2F). Moreover, the majority of the repressed genes interact with AT1s (Figure 2G). This suggests that *RET^C634Y^*-dependent signature repressed in NSCLC tumor samples may affect several distinct normal lung cell subpopulations through a general lung dedifferentiation program. The observation aligns with the finding that RET fusion-positive lung carcinomas displayed a higher prevalence of poorly differentiated tumors in comparison to those with ALK or EGFR alterations [11].

### 3.5. RET^C634Y^-Dependent Signature in NSCLC Is Associated with Poor Prognosis

**Figure 3:**
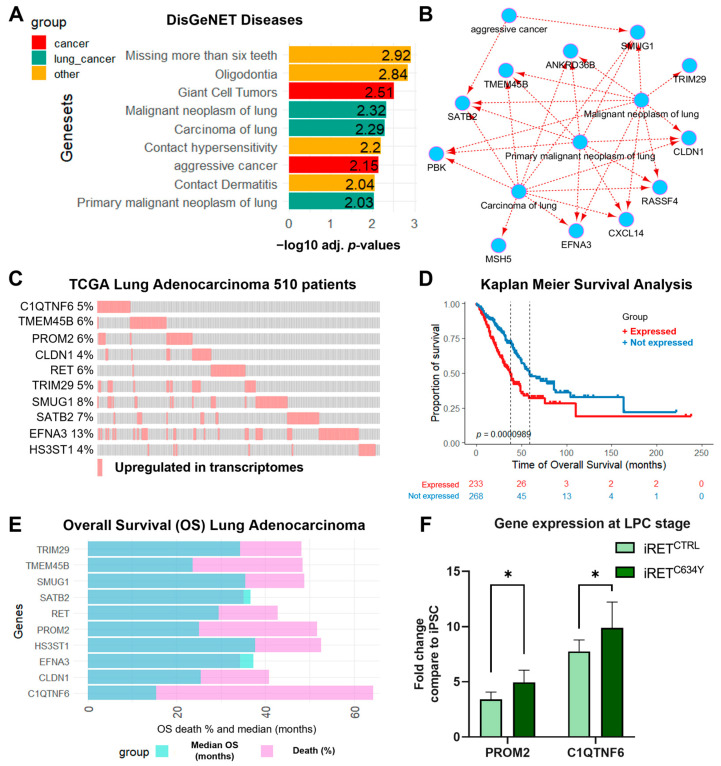
Adverse lung cancer prognosis for patients overexpressing *RET^C634Y^*-dependent activated signature. (**A**) Barplot of functional enrichment performed with *RET^C634Y^*-dependent activated signature on DisGeNET disease database. (**B**) Lung cancer related networks of genes found upregulated in NSCLC with *RET^C634Y^*-dependent activated model integration. (**C**) Oncoprint of the RET 10 genes signature in the transcriptome of the TCGA lung adenocarcinoma cohort (510 patients/510 samples). (**D**) Kaplan–Meier curve and log-rank test analysis assessing the overall survival (OS) of lung adenocarcinoma patients, comparing those with (red) and without (blue) the overexpression of RET 10 genes signature. (**E**) Barplot of univariate overall survival analysis for the individual genes of RET 10 genes signature. The proportion of patient deaths among those exhibiting gene overexpression (purple) and the corresponding median overall survival (blue) are displayed. (**F**) Expression of two cancer markers associated with adverse prognosis quantified by qRT-PCR. Fold changes (2^−ΔΔCt^) have been normalized to iPSC stage. Experiments were performed three times. *p*-values were calculated using a two-tailed Student’s *t*-test. * *p* < 0.05.

*RET^C634Y^*-dependent activated signature, constituted by the 33 genes found upregulated in NSCLC (Supplementary Table S3), was verified to stratify NSCLC tumor samples from normal lung by unsupervised clustering (Supplementary Figure S1C) but also by unsupervised PCA (Supplementary Figure S1D). Functional enrichment performed on DisGeNET database confirmed that *RET^C634Y^*-dependent activated signature is associated with known lung cancer pathogenesis such as carcinoma and malignant neoplasia (Figure 3A). Moreover, this signature can be integrated in a network of genes related to aggressive cancer signature (Figure 3B). These results suggest that *RET^C634Y^*-dependent activated signature could be associated with patients with a poor prognosis.

The transcriptome data obtained through RNA-sequencing from a cohort of 510 lung adenocarcinoma patients, compiled by The Cancer Genome Atlas (TCGA), were examined by comparing them to *RET^C634Y^*-dependent activated signature. *RET* and a subset of nine genes was found to be overexpressed in more than 4% of tumor samples (Figure 3C). Among these genes, six were already identified in the lung cancer related network of genes associated with *RET^C634Y^* signature (*TMEM45B*, *CLDN1*, *TRIM29*, *SMUG1*, *SATB2,* and *EFNA3*) (Figure 3B). The three other genes are *HS3ST1*, *PROM2,* and *C1QTNF6*. Combined overexpression of these nine genes with *RET* in TCGA lung cancer transcriptomes was found to significantly stratify patients according to their overall survival (Figure 3D). Moreover, univariate overall survival analysis of these individual 10 genes overexpressed in adenocarcinoma revealed a dramatic prognosis, the worst being *C1QTNF6* and *PROM2* overexpression (Figure 3E). The expression of *C1QTNF6* and *PROM2* was quantified by qRT-PCR and both genes were found to be upregulated in iRET^C634Y^ LPCs as compared to iRET^CTRL^ LPCs, indicating that *RET^C634Y^* is associated with the overexpression of NSCLC poor prognosis markers (Figure 3F).

The overexpression of this 10 gene signature can be significantly associated with the clinical data of patients (Supplementary Figure S2A). For example, an increase of hypoxia can be computed by three distinct scores (Winter, Ragnum, and Buffa scores) (Supplementary Figure S2B). A significant association was also observed with the increase of genomic alteration scores such as the fraction of genome altered, MSIsensor score, and tumor mutation burden (Supplementary Figure S2C). Other parameters reflecting the genomic instability such as the mutation count and the aneuploidy score were also found to be significantly increased with this signature (Supplementary Figure S2C). All together, these results confirmed that *RET^C634Y^*-dependent activated signature is associated with poor prognosis in lung cancer.

### 3.6. Differentiation of LPCs from RET^C634Y^ Knock-In iPSCs Results in the Overexpression of FOXA2 and NKX2-1

**Figure 4:**
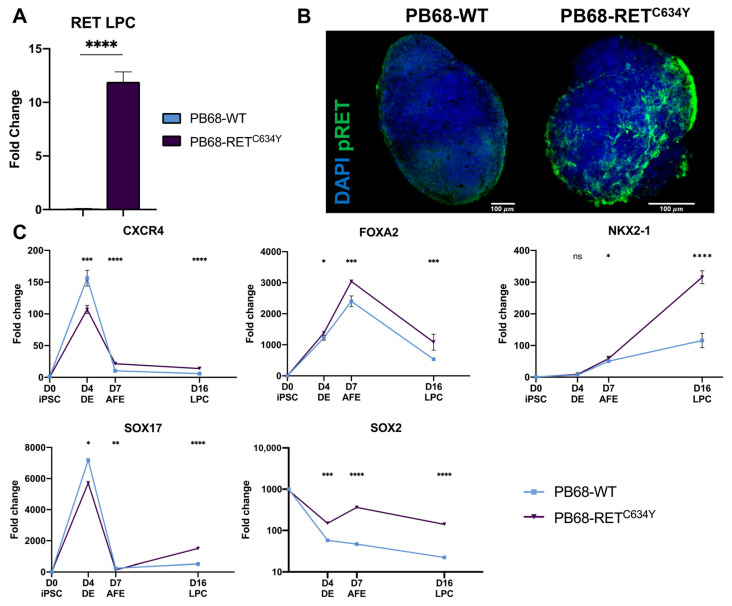
LPCs generated from *RET^C634Y^* knock-in iPSCs are also associated with an overexpression of FOXA2 and NKX2-1. (**A**) qRT-PCR quantification of RET mRNA in LPCs derived from PB68-WT and PB68-RET^C634Y^ iPSCs. (**B**) Immunostaining of LPCs derived from PB68-WT and PB68-RET^C634Y^ iPSCs showing the expression of pRET (green) and DAPI (blue). (**C**) Expression of the differentiation markers specific to each stage quantified by qRT-PCR. Fold changes (2^−ΔΔCt^) have been normalized to iPSC stage. Differentiation experiments were performed three times for each condition. *p*-values were calculated at each stage using a two-tailed Student’s *t*-test. ns, not significant; ns: non-significant, * *p* < 0.05; ** *p* < 0.01; *** *p* < 0.001; **** *p* < 0.0001.

To ascertain the impact of the *RET^C634Y^* mutation on LPC differentiation, a *RET^C634Y^* knock-in model (PB68-RET^C634Y^) was generated using a wild-type (WT) iPSC (PB68-WT) [33]. Using the previously described differentiation protocol, both iPSC lines were differentiated into LPCs. *RET* overexpression was confirmed through qRT-PCR analysis, revealing more than a 10-fold increase in expression in LPCs derived from PB68-RET^C634Y^ as compared to PB68-WT (Figure 4A). Activation of the RET pathway through its phosphorylation was assessed using immunofluorescence staining, which displayed a robust phospho-RET signal in LPCs derived from PB68-RET^C634Y^, whereas LPCs derived from PB68-WT showed minimal signal. (Figure 4B). These results confirmed the successful generation of a *RET^C634Y^* knock-in model by showing the overexpression and activation of RET in PB68-RET^C634Y^.

As described previously, qRT-PCR analysis was performed to assess the expression levels of stage-specific markers at various time points (Figure 4C). Strikingly, the knock-in of *RET^C634Y^* mutation exhibited a similar effect on the expression of the differentiation markers when compared to the patient-derived *RET^C634Y^* mutation. This effect was evident in the downregulation of *CXCR4* and *SOX17* during the DE stage, as well as the upregulation of *FOXA2* and *NKX2-1* during the AFE and LPC stages, respectively. However, it is noteworthy that the observed results for *SOX2* contrasted with the findings described earlier. Collectively, these findings strongly indicate a significant association between the *RET^C634Y^* mutation, and the observed phenotypes associated with NSCLC during LPC differentiation.

### 3.7. RET^C634Y^ Knock-In Induces a Signature of Fibroblastic and Metastatic Lung Adenocarcinoma in iPSC-Derived LPCs

**Figure 5:**
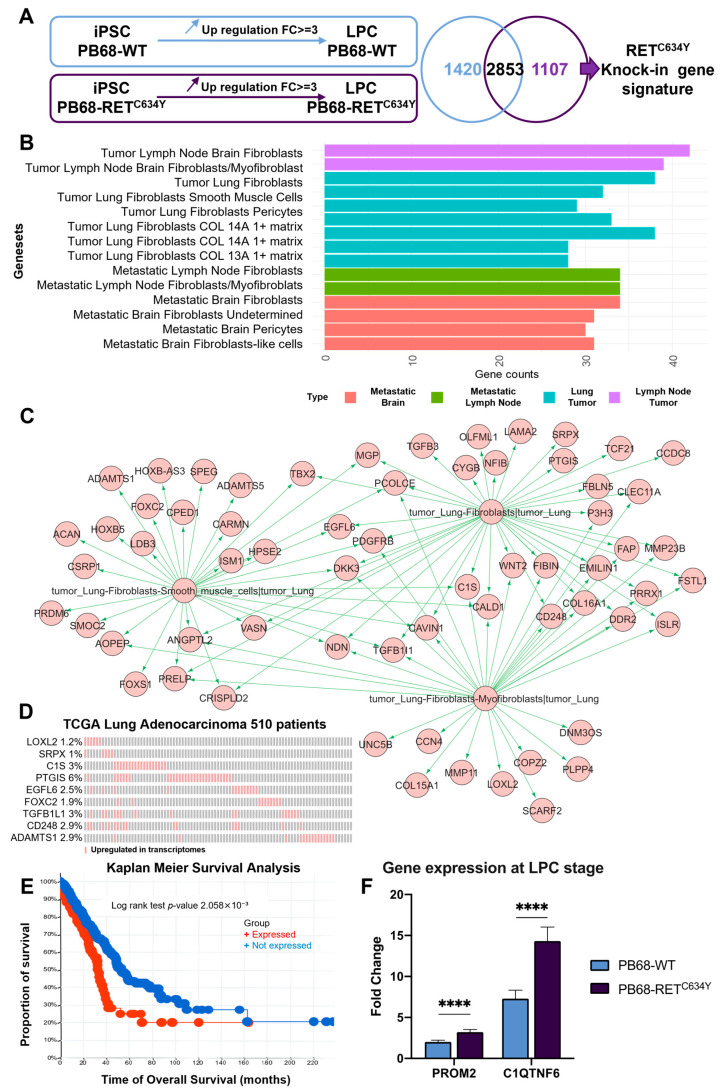
*RET^C634Y^* knock-in (RET-KI) induced a metastatic and fibroblastic lung adenocarcinoma expression signature in iPSC-derived LPCs. (**A**) Method for analyzing the RET-KI dependent signature during iPSC-derived LPC differentiation. (**B**) Functional enrichment on a single-cell atlas of metastatic lung adenocarcinoma with the genes upregulated specifically during RET-KI LPC differentiation. (**C**) Fibroblastic functional enriched network drawn during RET-KI LPC differentiation. (**D**) Oncoprint of RET-KI markers found overexpressed in the transcriptome of the TCGA lung adenocarcinoma cohort (510 patients/510 samples). (**E**) Kaplan–Meier curve and log-rank test analysis assessing the overall survival (OS) of lung adenocarcinoma patients, comparing those with (red) and without (blue) the overexpression of RET-KI markers. (**F**) Expression of C1QTNF6 and PROM2, two cancers markers associated with adverse prognosis, quantified by qRT-PCR at LPC stage. Fold changes (2^−ΔΔCt^) were normalized to iPSC stage. Experiments were performed three times. *p*-values were calculated using a two-tailed Student’s *t*-test. **** *p* < 0.0001.

To validate the impact of *RET^C634Y^* knock-in on transcriptional regulation during iPSC-derived LPC differentiation, whole transcriptome sequencing was done in triplicate for PB68-WT and PB68-RET^C634Y^ at both iPSC and LPC stages. The same analysis protocol employed for iRET iPSCs was applied, revealing a set of 1107 genes specifically overexpressed during PB68-RET^C634Y^ LPC differentiation. These genes constitute a specific *RET^C634Y^* knock-in (RET-KI) signature (Figure 5A). This specific RET-KI signature was used to perform functional enrichment analysis on a single-cell atlas of metastatic lung adenocarcinoma [61]. This analysis revealed a notable enrichment of these genes within the signature of fibroblastic and metastatic lung adenocarcinoma (Figure 5B). With this enrichment, it was possible to build a lung fibroblast related gene network which shared some markers with other subtypes of tumor microenvironment cells like myofibroblasts and smooth muscle cells (Figure 5C). Among the genes involved in this network, nine of them were found to be overexpressed in more than 1% of lung samples based on the TCGA 510 lung adenocarcinoma patient RNA sequencing (Figure 5D). Moreover, the combinatorial overexpression of these nine markers was found to be associated with unfavorable overall survival of the patients (Figure 5E). Moreover, crossing the *RET^C634Y^*-dependent gene signature with the specific RET-KI gene signature revealed 67 commonly regulated genes during these two experiments, including *C1QTNF6* (Supplementary Table S4). The expression of *C1QTNF6* and *PROM2* was also quantified by qRT-PCR and both genes were found to be in PB68-RET^C634Y^ LPCs as compared to PB68-WT LPCs indicating that RET pathway activation is associated with the overexpression of these two NSCLC poor prognosis markers (Figure 5F). Collectively, these data indicate that RET-KI induced a signature of metastatic lung adenocarcinoma during iPSC-derived LPC differentiation. Hence, this knock-in experiment serves to validate the role of the *RET^C634Y^*mutation as a driver of NSCLC features in LPCs derived from iPSCs carrying RET mutation.

### 3.8. RET Inhibitor Treatment Leads to the Downregulation of the Cancer Associated Marker in LPCs Derived from RET^C634Y^ iPSCs

To confirm that the observed phenotypes were specifically induced by the *RET^C634Y^*mutation during LPC differentiation, we added into the differentiation media the RET inhibitor pralsetinib (BLU-667) at a daily concentration of 10 nM. Pralsetinib is a drug designed to selectively target oncogenic RET alterations such as KIF5B-RET and CCDC6-RET fusions and *RET^C634Y^* mutations [62]. It is currently being tested in phase I/II of the ARROW trial and exhibits promising results [63].

**Figure 6:**
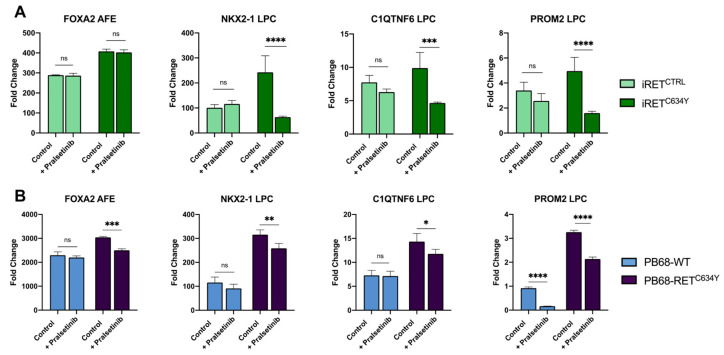
RET inhibitor pralsetinib treatment has a specific inhibitory effect on the genes upregulated by *RET^C634Y^*mutation. (**A**,**B**) Expression of *FOXA2*, *NKX2-1*, *C1QTNF6*, *PROM2* quantified by qRT-PCR in iRET model (**A**) and PB68 model (**B**) with and without daily 10 nM pralsetinib treatment. Fold changes (2^−ΔΔCt^) have been normalized to iPSC stage. Experiments were performed three times. Two-ways ANOVA was performed to test the effect of cell lines and pralsetinib treatment. For each combination of cell lines and genes, a Sidak’s multiple comparisons test was performed to test the effect of pralsetinib treatment as compared to WT. ns: non-significant, * *p* < 0.05; ** *p* < 0.01; *** *p* < 0.001; **** *p* < 0.0001.

Through qRT-PCR analysis, we measured the expression levels of the differentiation markers *FOXA2* and *NKX2-1*, which were found to be regulated by *RET^C634Y^* in our models, along with the cancerous markers *C1QTNF6* and *PROM2* previously identified. The gene expressions were measured on cells differentiated with and without pralsetinib. (Figure 6). In the iRET model, the expressions of *NKX2-1*, *C1QTNF6*, and *PROM2* exhibited highly significant levels of interactions between the cell lines and the two conditions (Supplementary Table S5). In all three cases, pairwise comparison analyses revealed that the addition of pralsetinib had no significant effect on LPCs derived from iRET^CTRL^ but led to a significant inhibition in the expression of the genes expressed by iRET^C634^ derived LPCs (Figure 6A). In the PB68 model, only *FOXA2* and *PROM2* genes exhibited significant levels of interactions between the cell lines and the two conditions but all four genes were affected significantly by pralsetinib treatment (Supplementary Table S6). Indeed, the addition of pralsetinib resulted in a significant inhibition of *FOXA2*, *NKX2-1*, and *C1QTNF6* in PB68-RET^C634Y^ cells, whereas no significant changes in expression were observed in PB68-WT cells (Figure 6B). Additionally, pralsetinib treatment led to a very strong inhibition of *PROM2* expression in all the cell lines (Figure 6B). In both models, a positive response to the RET inhibitor pralsetinib was observed as demonstrated by the downregulation of the cancer markers, indicating that their expression is regulated by *RET^C634Y^* mutation. Therefore, this validates the suitability of such models as valuable tools for testing potential drugs and for identifying new therapeutic options for RET-driven NSCLC treatment.

## 4. Discussion

*RET* rearrangements occur in approximately 1–2% of NSCLC, but they tend to affect a younger population of patients, and they are more frequently observed in individuals who have never smoked or have a limited smoking history [7]. *RET* rearrangements generate a novel fusion oncogene that leads to constitutive activation of the RET kinase domain [64]. This activation promotes downstream signaling pathways, such as the MAPK and PI3K-AKT pathways, which are critical for cell proliferation, survival, and other cancer-related processes [8,10]. *RET*-rearranged NSCLC lung cancers are also known to exhibit less differentiated tumors compared to other molecular types of NSCLC [11]. Furthermore, the *RET^C634Y^* mutation is a specific genetic alteration commonly found in medullary thyroid carcinoma (MTC). *RET^C634Y^* mutation leads to the constitutive activation of RET also resulting in uncontrolled cell growth and proliferation. Consequently, the effects of the *RET^C634Y^*mutation on the activation of the RET pathway resemble those seen in other *RET* rearrangements observed NSCLC [29,30,31].

Developing new models of NSCLC, particularly those involving rare oncogenic drivers such as RET, holds significant promise for advancing the development of novel therapies. Furthermore, the use of iPSCs to generate such models offers numerous advantages. They serve as an inexhaustible source of patient-specific cells, allowing researchers to investigate disease mechanisms, accelerate drug discovery, and explore the possibilities of personalized cell-based therapies with unprecedented potential [22,65]. However, the reprogramming of lung differentiated cells poses significant challenges, leading to the absence of NSCLC patient-derived iPSC lines and therefore the lack of NSCLC models derived from patient iPSCs. The objective of this study was to evaluate the viability of using iPSCs derived from patients carrying *RET* inherited mutations as an alternative method for developing RET-driven NSCLC models from iPSCs.

However, iPSCs exhibit unpredictable variability in their ability to differentiate into functional cells of a specific lineage due to their genetic background. This can pose challenges when comparing cells differentiated from patient-specific iPSCs and control iPSCs [66]. To address this issue and isolate the impact of oncogenic driver mutations, isogenic pairs of disease-specific and control iPSCs were generated [23,67]. The widespread adoption of genome editing tools, such as the CRISPR/Cas9 system, allows for the creation of control iPSCs, wherein the oncogenic mutation is corrected [68,69,70]. By differentiating in parallel patient-derived iPSCs and their CRISPR-corrected isogenic control iPSCs, it is possible to identify the distinct characteristics linked to the mutation. This approach was employed to evaluate the impact of *RET^C634Y^* mutation on the differentiation of LPCs from iPSCs by comparing patient-derived iPSCs carrying the *RET^C634Y^* mutation (iRET^C634Y^) with its CRISPR-corrected isogenic control (iRET^CTRL^) [34,35]. This strategy was complemented with the generation of a model of *RET^C634Y^*knock-in in an iPSC derived from a healthy donor (PB68-RET^C634Y^ and PB68-WT, respectively) [33]. The knock-in of a mutated gene in iPSCs has already been shown to successfully generate the NSCLC model. Indeed, Dost and colleagues showed that the introduction of *KRAS^G12D^* in healthy iPSC induces the development of NSCLC in iPSC-derived lung organoids [24].

Therefore, in this study, we employed these two approaches to successfully generate lung cell progenitors (LPCs) from iPSCs expressing the *RET^C634Y^* mutation (Figure 1A–C). We demonstrated that these LPCs exhibit several characteristics associated with *RET*-rearranged NSCLC when compared to control iPSCs (Figure 1 and Figure 4). For instance, *FOXA2* was found to be overexpressed at the AFE stage in both models by *RET* mutations, consistent with its upregulation by KIF5B-RET fusion in NSCLC through RET downstream signaling pathways (Figure 1E and Figure 4C) [55]. Furthermore, in both models the *RET^C634Y^* mutation was found to upregulate *NKX2-1*. NKX2-1 serves as a marker for lung progenitors, but it has also been identified as a tumor biomarker in lung cancer [57] due to its overexpression in adenocarcinoma [58,59].

Transcriptomic analyses performed on LPCs derived from the patient-derived iPSC iRET^C634Y^ and from its CRISPR-corrected control iRET^CTRL^, revealed a specific RET^C634Y^ signature (Figure 2A–C). We identified a subset of 10 genes, including *C1QTNF6* and *PROM2*, that showed a significant correlation with patients with poor prognosis (Figure 3C–E). *C1QTNF6* or C1q/tumor necrosis factor-related protein 6, is known to promote cell proliferation, migration, and invasion while inhibiting apoptosis in NSCLC, both in vitro and in vivo [71]. Additionally, *PROM2* overexpression is associated with poor overall survival in lung cancer [72]. However, these two cancerous markers have not yet been identified in *RET*-rearranged NSCLC. Studying the expression of these genes in patient samples could be valuable in assessing whether our models can predict adverse prognostic markers linked to *RET*-rearrangements and identify novel therapeutic targets. Particularly, considering that PROM2 is a membrane receptor, it could hold significant potential as a target for CAR-T cell therapy.

Additionally, a similar transcriptomic analysis was performed with the RET knock-in model to validate the impact of *RET^C634Y^* mutation on transcriptional regulation during iPSC-derived LPC differentiation (Figure 5). Whole transcriptome sequencing revealed a specific *RET^C634Y^* knock-in (RET-KI) signature associated with unfavorable overall survival in patients (Figure 5B–E). Additionally, 67 genes were commonly regulated in both *RET^C634Y^*-dependent and RET-KI signatures, including *C1QTNF6* (Supplementary Table S4). Subsequently, we confirmed the upregulation of *C1QTNF6* and *PROM2* through qRT-PCR analysis in the LPCs derived from the two models of *RET^C634Y^*-mutated iPSCs (Figure 3F and Figure 5F). This suggests that RET pathway activation is associated with the overexpression of these poor prognosis markers in both models of RET-driven NSCLC. Overall, this knock-in experiment validated the role of *RET^C634Y^* mutation as a driver of NSCLC features in LPCs derived from iPSCs.

*RET* rearrangements are considered as actionable molecular alteration, meaning they can be specifically targeted with precision medicines such as pralsetinib [63]. In our models of RET-driven NSCLC, LPCs derived from RET mutated iPSCs responded positively to the pralsetinib treatment (Figure 6). They showed a downregulation of *NKX2-1* and *FOXA2* as well as the cancerous markers *C1QTNF6* and *PROM2,* while almost no significant effect was observed on LPCs derived from control iPSCs (Figure 6A,B). These findings are interesting as they verify that the upregulation of these markers is indeed regulated by the RET pathway. Moreover, there is currently a lack of comprehensive investigation into the efficacy of RET inhibitors in preclinical lung cancer models with RET fusions [17]. The reported effectiveness of pralsetinib and other specific RET inhibitors such as cabozantinib or selpercatinib has been limited to only a few patient-derived lung cancer cell lines or PDX models [17,73]. The scarcity of patient-derived disease models is likely responsible for the limited available data in this area. Therefore, our model of patient-derived iPSCs could serve as a solution to this problem. However, while targeted therapies have shown promising results, acquired resistance to RET inhibitors can develop over time. Understanding the mechanisms of resistance is essential to develop strategies to overcome it and prolong the effectiveness of treatment. Our RET-driven NSCLC model derived from patient iPSCs offers the potential to generate iPSC clones resistant to pralsetinib treatment. These resistant clones can then be employed to study the underlying mechanisms of resistance or to identify new drugs that can effectively overcome this resistance.

While LPCs derived from *RET^C634Y^* iPSCs may not fully replicate all the characteristics of patients *RET*-rearranged NSCLC due to the involvement of complex processes and interactions between differentiated tissues, our study demonstrates that it serves as an accurate and easily generable model. With a 16-day differentiation protocol, we showed that this model can be used for drug testing and the identification of potential novel cancer biomarkers. At present, our focus is on generating mature 3D organoids from these LPCs, which we believe will enhance the accuracy of the *RET*-rearranged NSCLC modeling. One limitation of our model is that, in primary NSCLC, RET activation occurs through *RET* rearrangements rather than *RET* mutations. However, the consequences of both alterations involve the activation of RET signaling via phosphorylation. This suggests that our model will be of significant interest for further developments. While the expression of some genes identified as overexpressed in RET mutant cell lines has not been observed in NSCLC, our model showed a clear correlation of the expression of genes such as *C1QTNF6* and *PROM2* with primary patient transcriptome and survival.

Overall, these findings suggest that the presence of the *RET^C634Y^* mutation alone is enough to induce a phenotype resembling that of *RET*-rearranged NSCLC in LPCs generated from iPSCs. Consequently, this study demonstrates the potential of using iPSCs derived from patients carrying inherited mutations to model diseases in cases where patient iPSCs are not readily available or difficult to generate. This study establishes the first model of RET-driven NSCLC LPCs generated from patient-derived iPSCs.

## Data Availability

Data is contained within the article and Supplementary Material.

## Supplementary Materials

The following supporting information can be downloaded at: https://www.mdpi.com/article/10.3390/cells12242847/s1, Figure S1: (A) Expression heatmap (GSE44077) of the 97 *RET^C634Y^*-dependent repressed genes in NSCLC. (B) Unsupervised principal component analysis based on the 97 repressed *RET^C634Y^*-dependent genes in NSCLC tumors (GSE44077). (C) Expression heatmap (GSE44077) of the 33 upregulated *RET^C634Y^*-dependent genes in NSCLC tumors. (D) Unsupervised principal component analysis based on the 33 upregulated *RET^C634Y^*-dependent genes in NSCLC tumors (GSE44077). Figure S2: Significant clinical associations found with RET 10 gene signature in lung adenocarcinoma tumors from TCGA cohort: (A) Barplot of significant clinical parameters found associated with the over expression of the RET 10 genes signature in TCGA lung adenocarcinoma cohort. (B) Significant associations with three distinct hypoxia scores. (C) Significant associations with sample parameters. Table S1: Primers used for qRT-PCR. Table S2: *RET^C634Y^*-dependent repressed gene signature in NSCLC tumor. Table S3: *RET^C634Y^*-dependent activated gene signature in NSCLC tumor. Table S4: 67 commonly regulated genes in *RET^C634Y^*-dependent gene signature and RET-KI gene signature. Table S5: Two-way ANOVA analyzing the effect of the cells and pralsetinib treatment on the gene expression in iRET model. Percentage of total variation and *p*-value summary are shown for each gene. ns: non-significant * *p* < 0.05; ** *p* < 0.01; *** *p* < 0.001; **** *p* < 0.0001. Table S6: Two-way ANOVA analyzing the effect of the cells and pralsetinib treatment on the gene expression in PB68 model. Percentage of total variation and *p*-value summary are shown for each gene. ns: non-significant * *p* < 0.05; ** *p* < 0.01; *** *p* < 0.001; **** *p* < 0.0001.
