# Supplementary material for: Modeling RET-Rearranged Non-Small Cell Lung Cancer (NSCLC): Generation of Lung Progenitor Cells (LPCs) from Patient-Derived Induced Pluripotent Stem Cells (iPSCs)
Source: Cells. 2023 Dec 15;12(24):2847. doi: 10.3390/cells12242847 (PMC10742233; doi:10.3390/cells12242847)
Supplement: Supplementary file 1 [file cells-12-02847-s001.zip › cells-2734079-supplementary.pdf]

Supplementary material

Supplementary figures

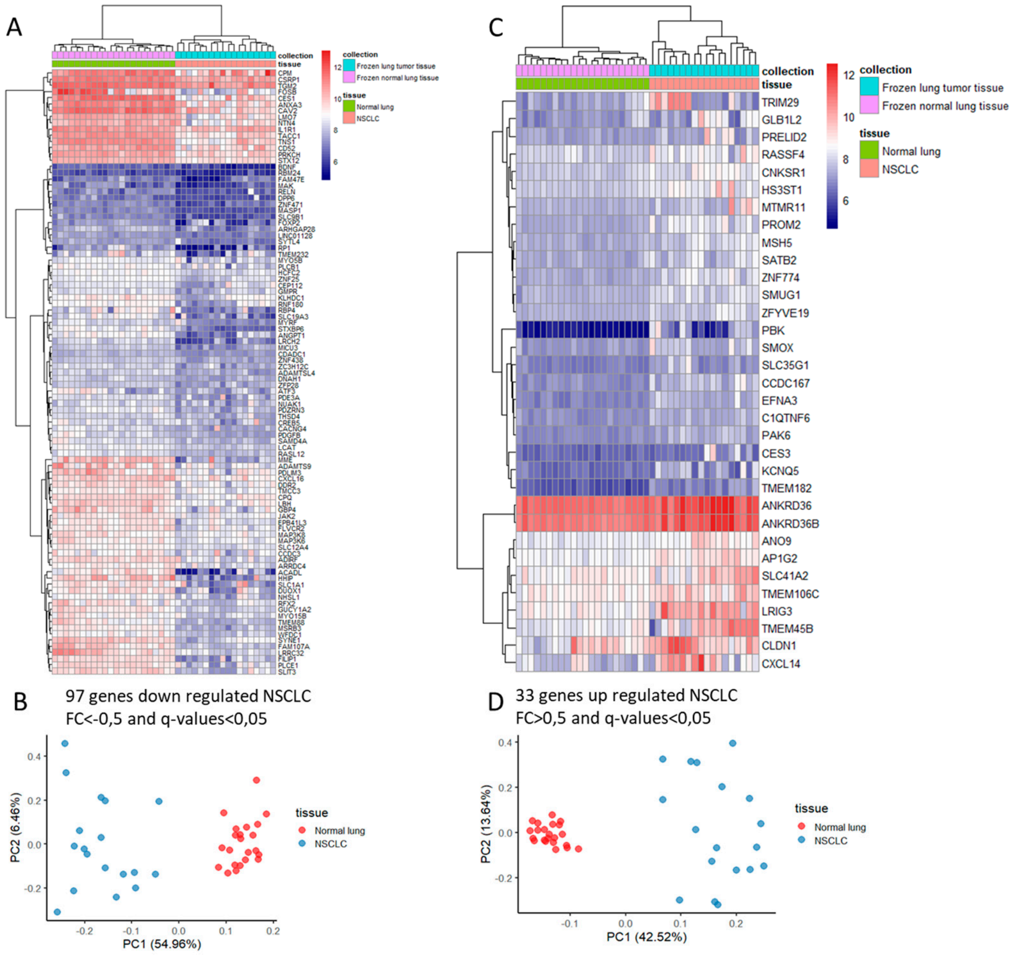

Supplemental Figure S1: (A) Expression heatmap (GSE44077) of the 97 RET^{C634Y}-dependent repressed genes in NSCLC tumors. **(B)** Unsupervised principal component analysis based on the 97 repressed RET^{C634Y}-dependent genes in NSCLC tumors (GSE44077). **(C)** Expression heatmap (GSE44077) of the 33 upregulated RET^{C634Y}-dependent genes in NSCLC tumors. **(D)** Unsupervised principal component analysis based on the 33 upregulated RET^{C634Y}-dependent genes in NSCLC tumors (GSE44077).

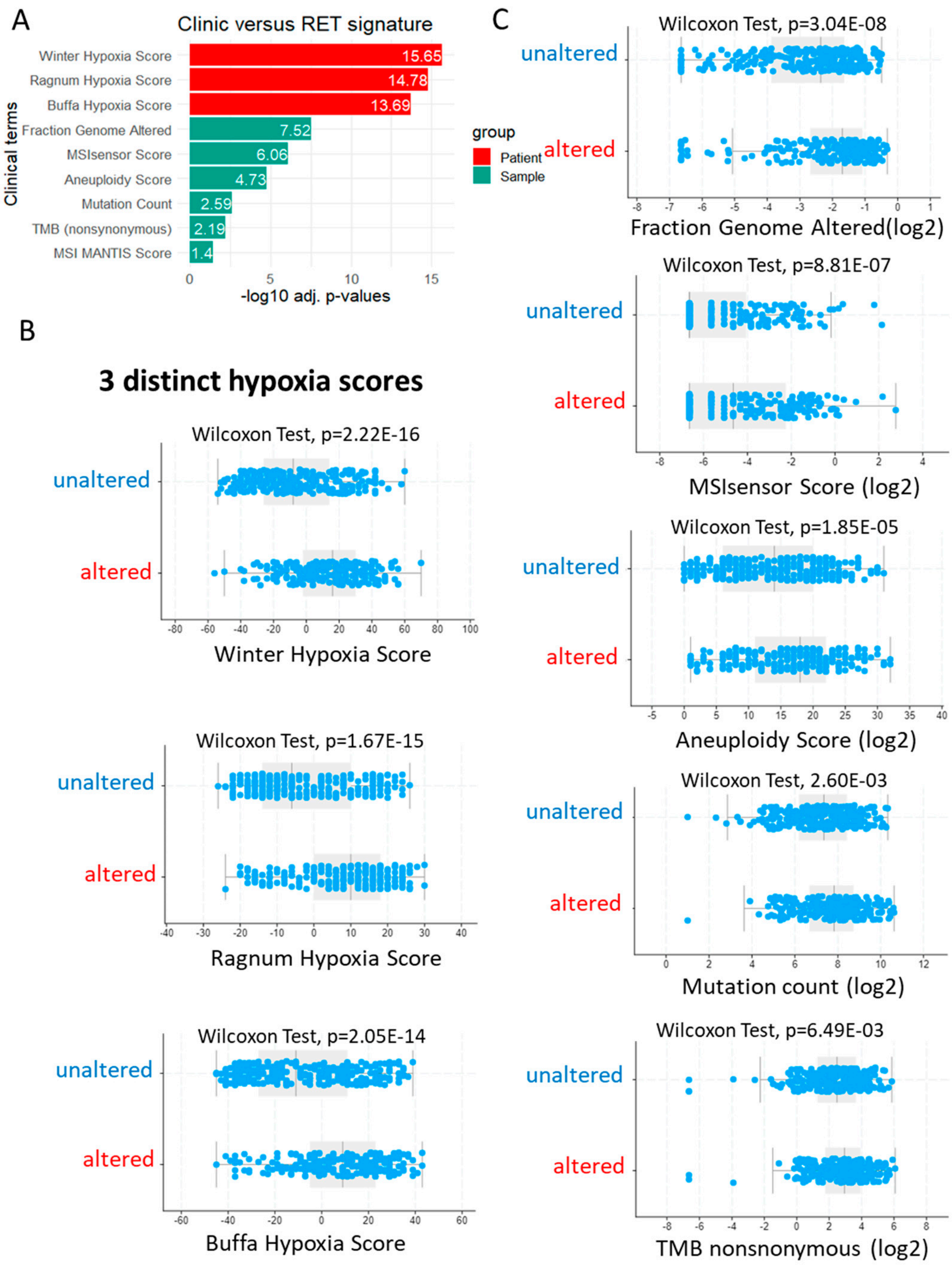

Supplemental Figure S2: Significant clinical associations found with RET 10 genes signature in lung adenocarcinoma tumors from TCGA cohort: (A) Barplot of significant clinical parameters found associated with the over expression of the RET 10 genes signature in TCGA lung adenocarcinoma cohort. **(B)** Significant associations with 3 distinct hypoxia score. **(C)** Significant associations with sample parameters.

Supplementary tables

Supplementary Table S1: Primers used for qRT-PCR

Primers	Sequence
ACTIN_FW	CACCATTGGCAATGAGCGGTTC
ACTIN_RV	AGGTCTTTGCGGATGTCCACGT
C1QTNF6_FW	GGTCAAGGGTTCTGTGAGGAG
C1QTNF6_RV	TCAGATGACTTTGGTGGAAGG

CXCR4_FW	CCCATCCTCTATGCTTTCCTTG
CXCR4_RV	GTCCACCTCGCTTTCCTTTG
FOXA2_FW	GCCGCAGATACCTCCTACTACC
FOXA2_RV	CCACTTGCTCTCTCACTTGTCC
NKX2-1_FW	CTCGCTCATTTGTTGGCGAC
NKX2-1_RV	CGTGTGCTTTGGACTCATCG
PROM2_FW	AGGCTGGAGAAGGATGTATGG
PROM2_RV	CAACTCTGAAGGGAAAGGATTG
RET_FW	CATCAGCAAAGACCTGGAGAAG
RET_RV	AATCAGGGAGTCAGATGGAGTG
SOX17_FW	CTGCAACTATCCTGACGTGTG
SOX17_RV	ACCCAGGAGTCTGAGGATTTC
SOX2_FW	CGAACCATCTCTGTGGTCTTG
SOX2_RV	ATTACCAACGGTGTCAACCTG

Supplementary Table S2: RET^{C634Y}-dependent repressed gene signature in NSCLC tumor

Gene	logFC	AveExpr	P.Value	adj.P.Val
ACADL	-2.799	8.126	7.87E-12	2.33E-10
HHIP	-2.608	8.820	5.85E-12	1.84E-10
MME	-2.442	9.065	1.24E-11	3.46E-10
FOSB	-2.250	9.783	7.15E-07	4.93E-06
CES1	-2.109	10.597	1.49E-13	8.32E-12
STXBP6	-2.023	7.788	1.28E-16	3.21E-14
FILIP1	-1.912	8.713	1.97E-12	7.07E-11
FAM107A	-1.875	8.948	1.05E-15	1.06E-13
ANGPT1	-1.748	8.079	5.36E-11	1.22E-09
LRRC32	-1.746	9.229	1.85E-11	4.90E-10
DUOX1	-1.741	8.731	4.89E-08	4.92E-07
SYNE1	-1.733	9.235	1.78E-14	1.12E-12
SLC19A3	-1.700	7.872	1.67E-08	1.78E-07
SLIT3	-1.668	8.911	3.78E-12	1.27E-10
CAV2	-1.642	10.792	6.66E-13	3.35E-11
LRCH2	-1.605	7.519	4.85E-11	1.16E-09
TNS1	-1.604	10.258	1.39E-12	5.81E-11
RBP4	-1.571	7.981	3.33E-06	1.78E-05
RP1	-1.566	6.732	2.33E-06	1.33E-05
SLC1A1	-1.559	8.681	1.11E-05	5.43E-05
MSRB3	-1.554	8.535	3.01E-18	1.52E-15
ANXA3	-1.490	10.456	1.03E-10	2.16E-09
TMEM88	-1.485	8.495	1.60E-12	6.18E-11
PLCE1	-1.447	8.952	2.44E-09	3.50E-08
LMO7	-1.385	9.901	3.35E-09	4.56E-08
GUCY1A2	-1.339	8.808	5.65E-16	9.48E-14
GBP4	-1.315	9.199	1.48E-06	8.96E-06
TACC1	-1.310	10.264	1.11E-14	7.96E-13
ADIRF	-1.261	9.117	2.16E-10	4.17E-09
ADAMTS9	-1.222	9.277	8.72E-06	4.34E-05
NTN4	-1.212	9.852	1.21E-07	1.15E-06
TMEM232	-1.189	7.778	8.61E-04	2.85E-03
PDLIM3	-1.175	9.387	1.28E-06	8.04E-06

RNF180	-1.129	8.480	3.46E-07	2.76E-06
RFX2	-1.108	8.774	8.04E-07	5.46E-06
WFDC1	-1.084	8.523	8.22E-11	1.80E-09
DDR2	-1.011	9.172	5.58E-09	6.52E-08
CD52	-1.008	10.210	8.76E-07	5.84E-06
EPB41L3	-1.007	8.948	4.70E-09	6.07E-08
MYRF	-1.006	7.804	1.34E-07	1.25E-06
NHSL1	-0.970	8.481	5.28E-06	2.77E-05
PDGFB	-0.952	8.020	1.13E-08	1.27E-07
LBH	-0.944	9.366	1.95E-09	2.98E-08
PRKCH	-0.943	10.184	3.35E-10	6.01E-09
PDZRN3	-0.913	8.164	1.45E-08	1.59E-07
KLHDC1	-0.902	8.846	2.79E-06	1.51E-05
MAK	-0.888	6.507	1.38E-05	6.60E-05
FLVCR2	-0.881	9.048	2.74E-07	2.22E-06
CPM	-0.869	10.868	1.84E-03	5.76E-03
SLC9B1	-0.860	6.647	5.21E-09	6.52E-08
MICU3	-0.853	7.697	2.41E-08	2.53E-07
MYO15B	-0.843	8.697	4.98E-07	3.48E-06
STX12	-0.823	9.987	5.43E-15	4.55E-13
MASP1	-0.817	6.646	9.41E-16	1.06E-13
ARRDC4	-0.794	8.844	8.82E-07	5.84E-06
PDE3A	-0.790	8.053	3.63E-04	1.30E-03
CPQ	-0.782	9.333	4.37E-07	3.21E-06
ARHGAP28	-0.770	7.450	1.68E-05	7.97E-05
ATF3	-0.759	8.029	3.96E-03	1.08E-02
CSRP1	-0.759	10.916	3.61E-10	6.27E-09
SAMD4A	-0.753	8.162	4.09E-07	3.07E-06
ZC3H12C	-0.751	7.988	9.82E-07	6.33E-06
FOXP2	-0.744	7.001	4.37E-03	1.16E-02
ADAMTSL4	-0.743	7.892	7.05E-09	8.06E-08
SLC12A4	-0.725	8.781	8.96E-13	4.10E-11
CACNG4	-0.725	8.033	1.82E-04	7.06E-04
CXCL16	-0.723	9.608	2.75E-06	1.51E-05
JAK2	-0.711	9.095	1.53E-07	1.33E-06
BDNF	-0.692	6.159	1.65E-06	9.88E-06
MAP3K8	-0.681	9.071	1.26E-06	8.04E-06
RASL12	-0.672	8.154	1.64E-10	3.31E-09
MAP3K6	-0.667	8.945	1.91E-06	1.12E-05
CCDC3	-0.655	8.783	4.08E-03	1.10E-02
FAM47E	-0.654	6.579	5.24E-03	1.34E-02
TGM2	-0.638	11.307	4.76E-07	3.39E-06
TMCC3	-0.636	9.015	1.41E-06	8.63E-06
LCAT	-0.629	8.397	7.96E-10	1.33E-08
PLCB1	-0.624	8.574	9.27E-04	3.03E-03
ZNF471	-0.595	6.774	7.83E-06	4.02E-05
CDADC1	-0.590	7.533	2.48E-10	4.61E-09
ZNF25	-0.590	8.482	1.89E-07	1.60E-06
SYTL4	-0.581	7.178	2.54E-05	1.16E-04
GMPR	-0.576	8.185	1.04E-04	4.23E-04
CREB5	-0.573	8.168	8.26E-03	1.98E-02
RELN	-0.559	6.735	3.58E-03	9.95E-03

CEP112	-0.550	8.285	3.92E-03	1.08E-02
DNAH1	-0.541	7.823	1.94E-06	1.12E-05
NUAK1	-0.533	8.024	2.60E-03	7.65E-03
HCFC2	-0.532	8.572	5.17E-08	5.10E-07
IL1R1	-0.520	10.581	8.56E-05	3.51E-04
ZNF438	-0.520	7.784	1.91E-07	1.60E-06
ZFP28	-0.518	7.814	8.09E-06	4.07E-05
RBM24	-0.514	5.990	2.35E-06	1.33E-05
LINC01128	-0.512	7.158	3.61E-07	2.83E-06
MYO5B	-0.511	8.388	6.60E-03	1.63E-02
DPP6	-0.509	6.565	3.43E-03	9.70E-03
THSD4	-0.509	8.041	3.56E-04	1.29E-03

Supplementary Table S3: RET^{C634Y}-dependent activated gene signature in NSCLC tumor

Gene	logFC	AveExpr	P.Value	adj.P.Val
PBK	1.666	5.811	2.51E-07	2.07E-06
CXCL14	1.559	8.459	3.22E-05	1.43E-04
LRIG3	1.327	9.242	4.04E-07	3.07E-06
CLDN1	1.140	9.410	5.31E-04	1.83E-03
TMEM45B	1.099	8.852	1.54E-03	4.87E-03
TRIM29	1.091	7.748	5.64E-03	1.43E-02
PROM2	0.845	7.650	1.51E-09	2.37E-08
CCDC167	0.843	7.215	4.41E-09	5.83E-08
KCNQ5	0.793	6.747	8.52E-05	3.51E-04
EFNA3	0.777	7.043	4.74E-11	1.16E-09
TMEM182	0.763	6.377	9.26E-10	1.50E-08
ZNF774	0.743	7.610	3.08E-09	4.30E-08
HS3ST1	0.701	8.090	6.98E-06	3.62E-05
AP1G2	0.685	8.693	5.44E-09	6.52E-08
SMOX	0.659	7.314	3.77E-05	1.64E-04
ANKRD36B	0.657	11.221	2.53E-06	1.41E-05
GLB1L2	0.654	7.373	1.12E-02	2.50E-02
MSH5	0.614	7.594	7.21E-08	6.97E-07
SLC41A2	0.585	9.029	6.35E-03	1.58E-02
ANO9	0.584	8.540	7.92E-06	4.02E-05
ANKRD36	0.583	11.413	1.93E-05	9.00E-05
PAK6	0.579	7.061	5.38E-09	6.52E-08
CES3	0.579	6.554	5.20E-03	1.34E-02
SMUG1	0.574	7.654	2.30E-09	3.41E-08
CNKSR1	0.573	8.019	4.78E-07	3.39E-06
MTMR11	0.570	7.669	8.80E-03	2.09E-02
TMEM106C	0.560	9.067	1.51E-04	6.04E-04
PRELID2	0.557	7.385	7.27E-03	1.77E-02
C1QTNF6	0.536	7.101	3.90E-08	4.01E-07
RASSF4	0.534	8.120	7.02E-04	2.37E-03
SLC35G1	0.531	6.861	2.95E-04	1.10E-03
SATB2	0.514	7.550	7.68E-05	3.24E-04
ZFYVE19	0.506	7.611	1.41E-07	1.25E-06

Supplementary Table S4: 67 commonly regulated genes in RET^{C634Y}-dependent gene signature and RET-KI gene signature

Gene symbol	Gene ID	Description
ACOX1	51	acyl-CoA oxidase 1
APBB3	10307	amyloid beta precursor protein binding family B member 3
ARHGAP5-AS1	84837	ARHGAP5 antisense RNA 1
ARPIN	348110	actin related protein 2/3 complex inhibitor
BTBD9	114781	BTB domain containing 9
C1QTNF6	114904	C1q and TNF related 6
C1S	716	complement C1s
CDC42EP1	11135	CDC42 effector protein 1
CEP112	201134	centrosomal protein 112
CHST3	9469	carbohydrate sulfotransferase 3
CPM	1368	carboxypeptidase M
CSRP1	1465	cysteine and glycine rich protein 1
CXCL1	2919	C-X-C motif chemokine ligand 1
DDR2	4921	discoidin domain receptor tyrosine kinase 2
EEF1AKMT3	25895	EEF1A lysine methyltransferase 3
ELK3	2004	ETS transcription factor ELK3
FILIP1	27145	filamin A interacting protein 1
GOLGA8O	728047	golgin A8 family member O
HCFC2	29915	host cell factor C2
HMGB1P31	100873894	high mobility group box 1 pseudogene 31
HS3ST1	9957	heparan sulfate-glucosamine 3-sulfotransferase 1
IL1R1	3554	interleukin 1 receptor type 1
IPP	3652	intracisternal A particle-promoted polypeptide
IRF9	10379	interferon regulatory factor 9
KANTR	102723508	KANTR integral membrane protein
KCNAB3	9196	potassium voltage-gated channel subfamily A regulatory beta subunit 3
KCTD21-AS1	100289388	KCTD21 antisense RNA 1
KIAA1841	NA	NA
LGALS8	3964	galectin 8
LINC00324	284029	long intergenic non-protein coding RNA 324
LINC01948	102467147	long intergenic non-protein coding RNA 1948
LRIG3	121227	leucine rich repeats and immunoglobulin like domains 3
LRRC32	2615	leucine rich repeat containing 32
LRTOMT	220074	leucine rich transmembrane and O-methyltransferase domain containing
LTBP3	4054	latent transforming growth factor beta binding protein 3
MAPK8IP2	23542	mitogen-activated protein kinase 8 interacting protein 2
MEGF6	1953	multiple EGF like domains 6
MICU3	286097	mitochondrial calcium uptake family member 3
MMP25-AS1	100507419	MMP25 antisense RNA 1
MTMR11	10903	myotubularin related protein 11
NCR3LG1	374383	natural killer cell cytotoxicity receptor 3 ligand 1
PDZRN3	23024	PDZ domain containing ring finger 3
PIGQ	9091	phosphatidylinositol glycan anchor biosynthesis class Q
RN7SL574P	106481079	RNA, 7SL, cytoplasmic 574, pseudogene
RNPEPL1	57140	arginyl aminopeptidase like 1
SCNN1D	6339	sodium channel epithelial 1 subunit delta
SEPTIN5	5413	septin 5

SLC35D1	23169	solute carrier family 35 member D1
SMPD3	55512	sphingomyelin phosphodiesterase 3
SOCAR	105373557	serous ovarian cancer associated RNA
SREBF1	6720	sterol regulatory element binding transcription factor 1
ST3GAL5	8869	ST3 beta-galactoside alpha-2,3-sialyltransferase 5
SUN2	25777	Sad1 and UNC84 domain containing 2
SYT17	51760	synaptotagmin 17
THNSL2	55258	threonine synthase like 2
THSD4	79875	thrombospondin type 1 domain containing 4
TMEM88	92162	transmembrane protein 88
TNS1	7145	tensin 1
TRAM2-AS1	401264	TRAM2 antisense RNA 1
TSPOAP1	9256	TSPO associated protein 1
USP47	55031	ubiquitin specific peptidase 47
ZBTB42	100128927	zinc finger and BTB domain containing 42
ZC3H12C	85463	zinc finger CCCH-type containing 12C
ZNF230	7773	zinc finger protein 230
ZNF292	23036	zinc finger protein 292
ZNF584	201514	zinc finger protein 584
ZNF688	146542	zinc finger protein 688

Supplementary Table S5: Two-way ANOVA analyzing the effect of the cells and Pralsetinib treatment on the gene expression in iRET model. Percentage of total variation and P-value summary are shown for each gene. ns: non-significant *, P<0.05; **, P< 0.01; ***, P<0.001; ****, P<0.0001.

iRET		Anova results		
Gene	Stade	Cell lines	Pralsetinib	Interraction
FOXA2	AFE	97,74% ****	0,52% ns	0,01% ns
NKX2-1	LPC	6,97% *	23,49% **	32,89% ***
C1QTNF6	LPC	0,28% ns	48,26% ***	15,15% *
PROM2	LPC	1,12% ns	59,01% ****	21,38% **

Supplementary Table S6: Two-way ANOVA analyzing the effect of the cells and Pralsetinib treatment on the gene expression in PB68 model. Percentage of total variation and P-value summary are shown for each gene. ns: non-significant *, P<0.05; **, P< 0.01; ***, P<0.001; ****, P<0.0001.

PB68		Anova results		
Gene	Stade	Cell lines	Pralsetinib	Interraction
FOXA2	AFE	62,30% ****	22,40% ****	10,86% **
NKX2-1	LPC	91,58% ****	4,50% **	0,68% ns
C1QTNF6	LPC	67,55% ****	3,64% ***	2,90% ns
PROM2	LPC	88,95% ****	16,93% ****	0,66% ***
